# Elucidation of the pathology and tissue distribution of *Lagovirus europaeus* GI.2/RHDV2 (rabbit haemorrhagic disease virus 2) in young and adult rabbits (*Oryctolagus cuniculus*)

**DOI:** 10.1186/s13567-018-0540-z

**Published:** 2018-06-05

**Authors:** Aleksija Neimanis, Ulrika Larsson Pettersson, Nina Huang, Dolores Gavier-Widén, Tanja Strive

**Affiliations:** 1 Department of Pathology and Wildlife Diseases, National Veterinary Institute (SVA), 751 89 Uppsala, Sweden; 2 Department of Biomedical Sciences and Veterinary Public Health, Swedish University of Agricultural Sciences (SLU), 750 07 Uppsala, Sweden; 3 Commonwealth Scientific and Industrial Research Organisation (CSIRO), Health & Biosecurity, Black Mountain Laboratories, Canberra, Australia

## Abstract

*Lagovirus europaeus* GI.2, also known as RHDV2 or RHDVb, is an emerging virus that causes rabbit haemorrhagic disease (RHD) in European rabbits (*Oryctolagus cuniculus*). In contrast to *L. europaeus* GI.1 (or RHDV/RHDVa) viruses that are only pathogenic for adults, GI.2 causes clinical disease in both adults and kittens. However, detailed descriptions of the pathology of this virus that may provide insight into its pathogenicity and emergence are lacking. Using an Australian GI.2 field strain isolated in 2015, we provide the first detailed description of pathology, viral antigen distribution and tissue load of GI.2 in adult and 5-week old New Zealand white rabbits using histology, immunohistochemistry and RT-qPCR. Liver was the target organ, but in contrast to GI.1 viruses, lesions and inflammatory responses did not differ between adults and kittens. Lymphocytic inflammation, proposed to be protective in kittens infected with GI.1, was notably absent. We also present the first descriptions of bone marrow changes in RHD, including decreased myeloid-to-erythroid ratio. Consistent with other pathogenic lagoviruses, intracellular viral antigen was demonstrated in hepatocytes and cells of the mononuclear phagocytic system. In terminal stages of disease, viral loads were highest in liver, serum and spleen. Despite the small sample size, our data suggest that unlike early European GI.2 strains, the pathogenicity of the Australian GI.2 virus is similar to GI.1 viruses. Additionally, GI.2 was fatal for all (*n* = 5) inoculated kittens in this study. This may significantly alter RHD epidemiology in the field, and may impact biocontrol programs for invasive rabbits in Australia where GI.1 viruses are intentionally released.

## Introduction

Rabbit haemorrhagic disease (RHD) is a viral disease of the European rabbit (*Oryctolagus cuniculus*) that primarily affects the liver [1]. The causative agents, currently known as rabbit haemorrhagic disease virus (RHDV), the antigenic variant (RHDVa), and the recently described RHDV2 or RHDVb, are lagoviruses within the Family *Caliciviridae* [1, 2]. Within a proposed new classification system [3], all lagoviruses are reclassified into a single species, *Lagovirus europaeus*, and the causative viruses of RHD are all grouped within genogroup I (*L. europaeus* GI) (Table 1).

**Table 1:** **A list of current names and proposed new, phylogenetically derived names for lagoviruses according to Le Pendu et al. [** 3 **] referred to in this manuscript**

Current name	Proposed new name [3]
Rabbit haemorrhagic disease virus (RHDV)	*Lagovirus europaeus* GI.1
RHDVa	*Lagovirus europaeus* GI.1a
RHDV G1	*Lagovirus europaeus* GI.1b
RHDV2 or RHDVb	*Lagovirus europaeus* GI.2
Recombinant of RHDV G1 and RHDV2, recombination break point between the polymerase and capsid coding regions	*Lagovirus europaeus* GI.1bP–GI.2

First described in domestic rabbits in China in 1984, RHD was detected in Europe in 1986 and has been documented at some point on every continent except Antarctica [1]. In Australia and New Zealand, RHD is used intentionally for biocontrol of invasive, introduced European rabbits. Until 2010, the disease was almost exclusively confined to wild and domestic European rabbits and was caused by viruses newly classified as genotype 1 of the *L. europaeus* GI group (i.e. *L. europaeus* GI.1 [3]), also known as RHDV and RHDVa (Table 1). *L. europaeus* GI.1 is further subdivided into variants denoted by lower case letters [3] (Table 1).

In 2010, a new genotype (*L. europaeus* GI.2 [3], also known as RHDV2 or RHDVb, Table 1) was detected in domestic and wild European rabbits in France [4]. It soon was reported throughout Europe [2, 5–8] and reached Australia in 2015 [9], where it rapidly spread and appears to be replacing previously endemic GI.1 strains [10] as has been described in some European countries [2, 11–15]. While disease mimics the disease caused by *L. europaeus* GI.1 viruses, disease course and levels of pathogenicity appeared to be more variable [2]. In addition, while animals younger than 5–8 weeks of age are highly resistant to the development of clinical disease following infection with *L. europaeus* GI.1 viruses, kittens as young as 11 days of age can succumb to disease and death from infection with *L. europaeus* GI.2 [13]. Finally, species barriers are less rigid within the Leporidae family, as *L. europaeus* GI.2 viruses can cause clinical disease and death in various hare species [16–20].

While there are numerous descriptions of the pathology resulting from infection by *L. europaeus* GI.1 (reviewed in [1]), to date, there are only a few, brief descriptions for *L. europaeus* GI.2 [2, 5, 11]. Additionally, some disparities regarding pathology and pathogenesis of disease caused by *L. europaeus* GI.1 have been reported. While many likely stem from differences in methodology as suggested by Abrantes et al. [1], differences between viral strains and hosts may also play a role. Finally, there appear to be strain differences in the type of disease caused by *L. europaeus* GI.2. Le Gall-Reculé et al. [2] report that early strains often resulted in subacute to chronic disease, whereas experimental work by Capucci et al. [21] suggests that more recent *L. europaeus* GI.2 strains result in predominantly acute disease. Detailed descriptions of disease caused by different *L. europaeus* GI.2 viruses therefore are warranted and may provide insight into mechanisms responsible for pathogenicity.

Presently, lagoviruses cannot be cultured ex vivo and experimental infections still are required to study certain aspects of disease and generate material for further analyses, such as viral antigen for serological assays. For this study, rabbits were infected with a *L. europaeus* GI.2 strain detected in Australia that is also closely related to circulating strains in southern Europe [22] and high quality material from both adults and kittens was collected to describe the pathology and tissue distribution of this virus in detail. We compare findings between the two age groups and with previously published findings for GI.1 strains to provide further insight into pathogenesis. We also interpret our findings in conjunction with parallel quantitative molecular analyses to evaluate virus localization and detection limits of our immunohistochemistry analyses.

## Materials and methods

The virus used in this study was the first *L. europaeus* GI.2 virus reported in Australia [9] (GenBank# KT280060). From here on this virus is referred to as BlMt-1, the name of this isolate. BlMt-1 is closely related to isolates described in the Iberian Peninsula [22] (>97% genetic identity), and is a recombinant virus with the capsid gene of GI.2 and the non-structural genes of GI.1 (GI.1bP–GI.2, according to the newly proposed nomenclature by Le Pendu et al. [3]) (Table 1).

Rabbits used in this study were sourced from the domestic rabbit breeding colony at CSIRO Black Mountain. Rabbits in this research colony are regularly tested and confirmed to be free of antibodies to RHDV as well as the non-pathogenic lagovirus RCV-A1 [23, 24] that is endemic to Australia and can attenuate RHDV infections [25]. All procedures involving live animals were carried out in Australia and were in accordance with the Australian Code of Practice for the Care and Use of Animals for Scientific Purposes (2013) and were approved by the CSIRO Ecosystem Sciences Animal Ethics Committee (permits ESAEC 13-10, DOMRAB).

To generate material for further research, two adult New Zealand white rabbits were inoculated per os with 1 mL of a clarified 2% w/v liver homogenate of BlMt-1 containing 3 × 10^9^ copies/mL. Although the infectivity of this preparation was not experimentally determined in rabbits, this likely constitutes a very high infectious dose. Rabbits were monitored twice daily or more frequently when required. Both animals were euthanized when they displayed signs of RHD, at 54 and 96 h post-inoculation (hpi) (Table 2). Humane endpoints for terminal RHD were defined as hypothermia (normal rectal temperature is defined as 38.5–40 °C) after a fever episode (>40.4 °C), depression with loss of muscle tone and/or anorexia and >10% acute weight loss. For euthanasia, rabbits were first sedated with an intramuscular injection of Ketamine/Xylazine (30; 5 mg/kg) (Xylazil-20: Troy Laboratories, Smithfield, Australia; Ketamav 100: Mavlab, Logan, Australia), followed by intravenous injection of pentobarbitone sodium (165 mg/kg) (Lethabarb: Virbac, Milperra, Australia) in the marginal ear vein. Blood collection and post-mortem examinations were performed immediately following euthanasia and the following suite of tissues was collected: liver (hilar region and distal lobe), gall bladder with bile, spleen, lung, heart, kidney, thymus, mesenteric lymph node, duodenum, jejunum, ileum, Peyer’s patches, appendix, sacculus rotundus, cecum, colon, trachea, femoral bone marrow and brain. The bone marrow was removed from the femur before placement in formalin to eliminate the need for decalcification before processing for microscopic analysis. Each sample was split in two. One half was fixed in 10% neutral buffered formalin (NBF) while the other was frozen at −20 °C for molecular analyses. A healthy adult rabbit from the same rabbit colony was killed humanely as described above to serve as a negative control and the same suite of samples were collected.

**Table 2:** **Domestic rabbits (** ***Oryctolagus cuniculus*** **) inoculated per os with** ***Lagovirus europaeus*** **GI.2 (BlMt-1)**

Animal identification number	Age	Euthanized (E) or died spontaneously (D)	Time (hours post infection) of euthanasia or death	Tissues for molecular analysis collected (Y = yes, N = no)	Tissues for microscopic analyses collected (Y = yes, N = no)
282	Adult	E	96	Y	Y
283	Adult	E	54	Y	Y
302	Juvenile	D	<90^a^	Y	N
303	Juvenile	D	66	Y	Y
304	Juvenile	D	<66^a^	Y	N
305	Juvenile	D	<66^a^	Y	N
306	Juvenile	E	75	Y	Y

Rabbit 130 (not shown), a healthy adult from the same research colony, was euthanized to serve as a negative control.

^a^ Rabbit was found dead.

Blood samples were collected by cardiac puncture following euthanasia and centrifuged at 3000 *g* for 20 min to separate the serum. White blood cells (buffy coat) were collected from the interphase and both serum and white blood cells were stored at −20 °C until analysis.

Young animals are resistant to the development of clinical disease following infection with *L. europaeus* GI.1 viruses [1]. To investigate the susceptibility of young animals to *L. europaeus* GI.2, five 5-week old New Zealand white rabbit kittens from the same rabbit colony were inoculated per os with 0.5 mL of a clarified liver homogenate of BlMt-1. Again, all died or were euthanized because of terminal signs of RHD within 90 h of inoculation (Table 2). One kitten that was euthanized and one that died while euthanasia was about to be performed were examined immediately after death and blood and the following subset of tissues were collected: liver, spleen, lung, heart, kidney, duodenum, jejunum, ileum, Peyer’s patches, appendix and colon. Again, half of each sample was placed in 10% NBF and half was frozen at −20 °C. The three other kittens were found dead. Exact time of death was not known. The same subset of tissues was collected, but samples were saved at −20 °C only. Samples for microscopic examination were not collected.

Viral load (capsid genome copies) was determined in all tissue samples collected, including bile, serum and buffy coat. Samples were analysed as described previously using a universal lagovirus quantitative reverse transcriptase PCR (RT-qPCR) assay that detects all lagoviruses known to be present in Australia [26].

Following formalin fixation for 1 week, tissues were processed and embedded in paraffin at the Imaging and Cytometry facility, Australian National University for routine microscopic examination.

All microscopic analyses, including immunohistochemistry, were performed at the National Veterinary Institute in Sweden. To assess histopathology, sections (3–4 µm) were stained with Mayer’s haematoxylin and eosin [27].

In order to perform a relative comparison of proportions of different cell lines in the bone marrow of the two infected adult rabbits and the control animal, estimates of the myeloid-to-erythroid ratio (M:E) and the proportion of early to later stage myeloid cells were determined. Analyses were conducted on formalin-fixed bone marrow stained with haematoxylin and eosin as described above. M:E ratios were calculated by classifying 300 nucleated cells at high power (100 cells per three different fields at 1000× magnification) into cell types and then dividing the number of myeloid lineage cells by the number of nucleated erythroid cells. Additionally, the proportion of early myeloid cells (myeloblasts, promyelocytes and myelocytes as defined by Riedel et al. [28]) in relation to later stage myeloid cells was estimated. This was done by classifying 200 myeloid cells (100 cells per two different fields at 1000× magnification) as early [large cells with round to oval nucleus, and at least one nucleolus (myeloblast) or eosinophilic cytoplasmic granules (promyelocyte and myelocyte)] or later stage myeloid cells (kidney bean-shaped, band-shaped or segmented nucleus and eosinophilic cytoplasmic granules).

To complement molecular analyses and provide further insight into virus localization, distribution of GI.2/RHDV2 viral antigen in situ, and its relation to presence of histological lesions, immunohistochemical staining was carried out on all formalin-fixed and paraffin-embedded tissues available from the four infected animals (282, 283, 303 and 306) and the control animal (130). Briefly, 3–4 µm sections were deparaffinized and rehydrated. Slides were then placed in an automated AutostainerPlus (Agilent Technologies Sweden AB, Kista, Sweden). Endogenous peroxide activity was blocked with hydrogen peroxide (Dako REAL™ Peroxidase-Blocking Solution, Agilent Technologies Sweden AB, Kista, Sweden) for 5 min. Sections were then treated with 2% bovine serum albumin (BSA) for 20 min. Slides were then incubated for 30 min at room temperature with a 1:700 dilution (diluted with Dako Antibody Diluent with Background Reducing Components, Agilent Technologies Sweden AB, Kista, Sweden) of a primary monoclonal antibody cocktail (3H6 + 6G2 IgG1 mouse monoclonal antibodies, concentration of 0.32 mg/mL) targeting the capsid protein of lagoviruses [29] kindly provided by the OIE Reference Laboratory for RHD, Brescia, Italy. Visualization of bound antibodies was performed by using the polymer detection system Dako EnVision+ System-HRP Labelled Polymer (anti-mouse) (Agilent Technologies Sweden AB, Kista, Sweden) for 30 min followed by application of the chromogen diaminobenzidine (DAB) (Dako Liquid DAB+ Substrate Chromogen System, Agilent Technologies Sweden AB, Kista, Sweden) for 10 min. Sections were then counterstained with Mayer’s haematoxylin for 5 min. For the immunoglobulin negative control, a duplicate section was incubated with a non-specific antibody (Negative Control Mouse IgG1, Agilent Technologies Sweden AB, Kista, Sweden) with the same isotype as the primary antibody, diluted to the same protein concentration as the primary antibody. A positive tissue control (liver from a wild rabbit in Sweden confirmed positive for *L. europaeus* GI.2 by RT-qPCR, sequencing and immunological characterization [8] and a negative tissue control (liver from a healthy, adult rabbit from the same Australian colony) were included on each slide to ensure immunolabelling was working for each slide and consistent among slides.

## Results

### Animals/clinical signs

All inoculated rabbits developed a fever (temperature > 40.4 °C) between 30 and 52 hpi. Rabbits 283 (an adult), 303 and 306 (both kittens) showed signs of terminal RHD (depressed, little resistance to handling) and were euthanized or died just before euthanasia could be carried out. Both 303 and 306 also had subnormal body temperature (< 38 °C). Rabbit 282 was active and behaving normally, but had lost > 10% of its body weight at 96 hpi and was euthanized. Rabbits 304, 305 and 302 were found dead. Time of death or euthanasia are summarized in Table 2.

### Gross pathology

Rabbit 282 had a tan and mottled liver, enlarged spleen and congested lungs. The tissues of rabbit 283 were unremarkable, except for an enlarged spleen.

All kittens had a tan and friable liver, and the liver was severely discoloured yellow-tan in rabbit 306. Rabbit 302 showed subserosal petechial haemorrhages over Peyer’s patches and multifocal haemorrhages around the mesenteric lymph nodes. Rabbit 304 had copious foamy nasal discharge, and rabbit 305 had hay in the mouth, indicating peracute death while eating. Tissues were otherwise unremarkable.

### Histopathology

There was a clear difference in severity and distribution of lesions from the adult euthanized at 54 hpi (rabbit 283) and the other three rabbits that were euthanized or died later (rabbits 282, 303 and 306, Table 2). Significant lesions were seen in the liver, spleen and bone marrow of all animals examined histologically, but these were less severe in rabbit 283. Additional lesions were observed in kidneys, lungs, heart and other lymphoid tissues of some, but not all, rabbits.

**Figure 1:**
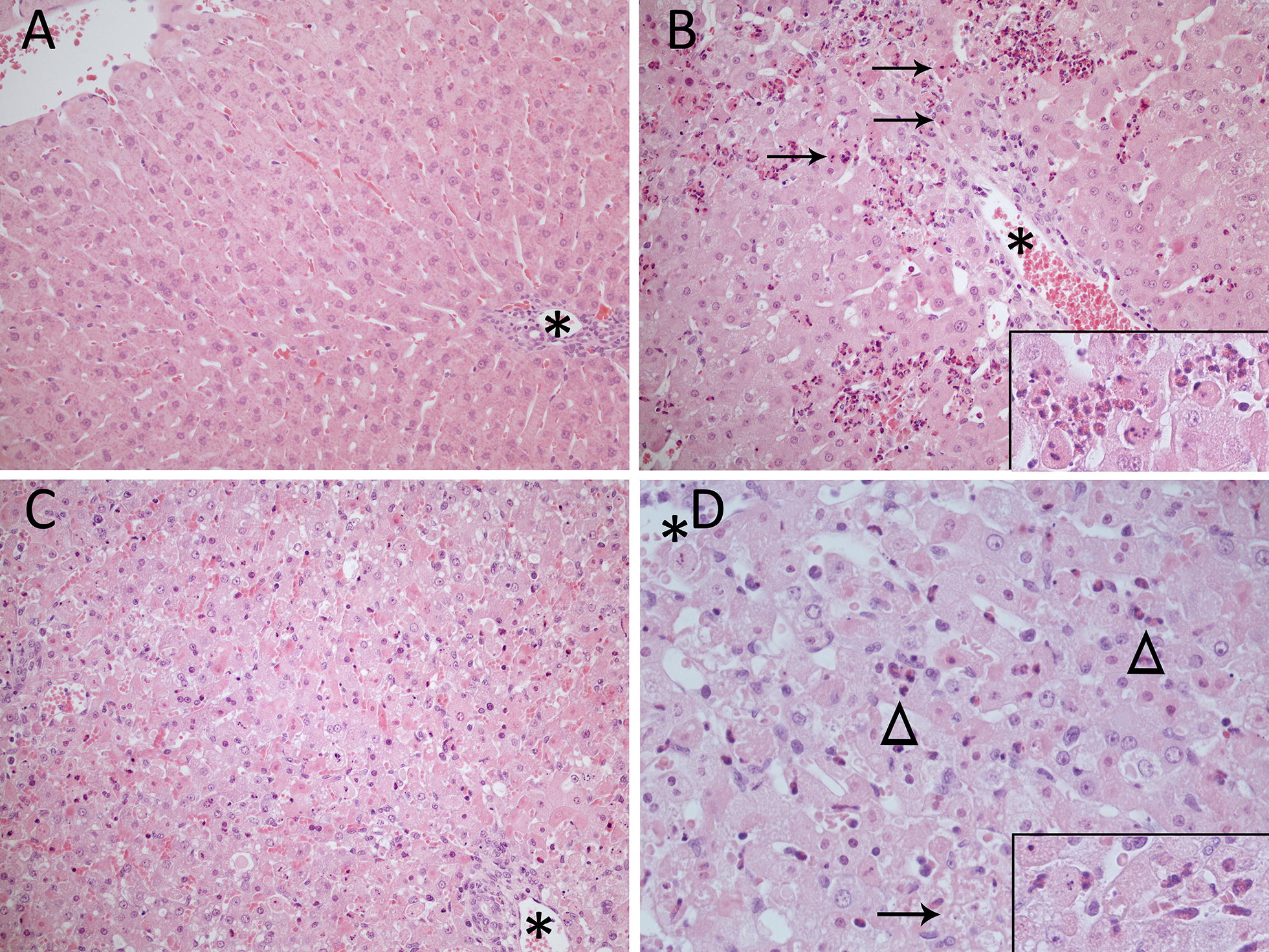
**Microscopic lesions in the liver of rabbits (*Oryctolagus cuniculus*) inoculated with** ***Lagovirus europaeus*** **G1.2 (BlMt-1).** Asterisks denote portal areas. Haematoxylin and eosin stain. **A** Uninfected control rabbit (rabbit 130). 200× magnification. **B** Earlier stage of acute infection in an adult rabbit (rabbit 283). There is degeneration and death of hepatocytes at the limiting plate and periportally surrounded by intensive heterophilic inflammation. Arrows denote apoptotic cells. 200× magnification. Insert: high magnification of dead hepatocytes surrounded by heterophils. **C** Terminal stage of acute infection in a 5 week old rabbit (rabbit 303). There is massive hepatocellular degeneration and death. 200× magnification. **D** Terminal stage of acute infection in a 5 week old rabbit (rabbit 306). Arrowheads denote clusters of heterophils that are seen throughout the lobule and the arrow indicates an area of lytic necrosis. 400× magnification. Insert: high magnification of heterophils surrounding dead hepatocytes.

Liver lesions of rabbit 283 were primarily in periportal areas. Mild to moderate numbers of single or small groups of periportal hepatocytes, including occasional cells of the limiting plate, were degenerate or dead and usually were surrounded and infiltrated by moderate to large numbers of heterophils (Figure 1). Occasional heterophils also infiltrated portal areas and were seen in sinusoids. Affected hepatocytes had hypereosinophilic cytoplasm, typically accompanied by a karyorrhectic or pyknotic nucleus. In advanced stages, cells or cell fragments had rounded up, were diminished in size, often contained pyknotic nuclear fragments, and had detached from hepatic cords (consistent with apoptotic cells and apopotic bodies, Figure 1). Less frequently, affected hypereosinophilic hepatocytes retained their shape and nuclei were faded (karyolysis). In other areas, heterophils were seen amongst fragmented remnants of hepatocytes, and where heterophil aggregates were more extensive, hepatocytes were effaced, resulting in focal lytic necrosis and sometimes haemorrhage. Occasional hepatocytes in midzonal regions were also affected but hepatocytes around central veins were almost entirely spared. Fine cytoplasmic vacuolation and mild to moderate small, distinct, round intracytoplasmic vacuoles were seen predominantly in midzonal hepatocytes and occasionally, an aggregate of hyaline, eosinophilic material was seen adjacent to the nucleus. Kupffer cells were mildly enlarged and contained a larger nucleus with an open chromatin pattern, resulting in these cells being more prominent than in the control animal. Bile ducts and vessels were unremarkable and no thrombi were seen. Similar to the control rabbit, portal areas also displayed mild to moderate bile duct hyperplasia, infiltration of scattered lymphocytes and plasma cells and mild, early fibrosis.

In the liver of the other three rabbits, hepatocytes showed the same changes indicative of degeneration and cell death as described for 283, but the extent and degree was much more severe (Figure 1). Although there was a tendency for periportal hepatocytes to be targeted, including hepatocytes of the limiting plate, affected hepatocytes could be seen throughout the lobule. The extent was so severe in the two kittens that cell death was classified as massive (Figure 1). Karyorrhexis was still common, but nuclei undergoing karyolysis were much more frequent than in rabbit 283. Round, hypereosinophilic hepatocellular remnants with or without pyknotic nuclear fragments were occasionally to frequently observed and individual dead cells that had lost cellular detail or were swollen and vacuolated instead of shrunken were also seen (more consistent with single cell necrosis as described in Elmore et al. [30]). While occasional (303) to moderate numbers of heterophils (282 and 306) were present, they were dispersed throughout sinusoids and infiltrated hepatic cords rather than being intensively aggregated periportally (Figure 1). Occasionally, they clearly surrounded dead hepatocytes. Small, scattered foci of lytic necrosis and haemorrhage also were seen. Hepatocytes throughout the lobule often contained distinct, round intracytoplasmic vacuoles consistent with lipid. Kupffer cells were plump, prominent and slightly to moderately more numerous compared to the control animal. In one kitten, fibrin thrombi were very occasionally seen in portal vessels, but in general, with the exception of occasional plump, activated endothelium, vessels were unremarkable. Like the control animal and 283, there was mild (kittens) to moderate (282) bile duct hyperplasia, mild, early fibrosis and infiltration of scattered lymphocytes and plasma cells in portal areas. Biliary epithelium and bile ducts showed no evidence of necrosis.

**Figure 2:**
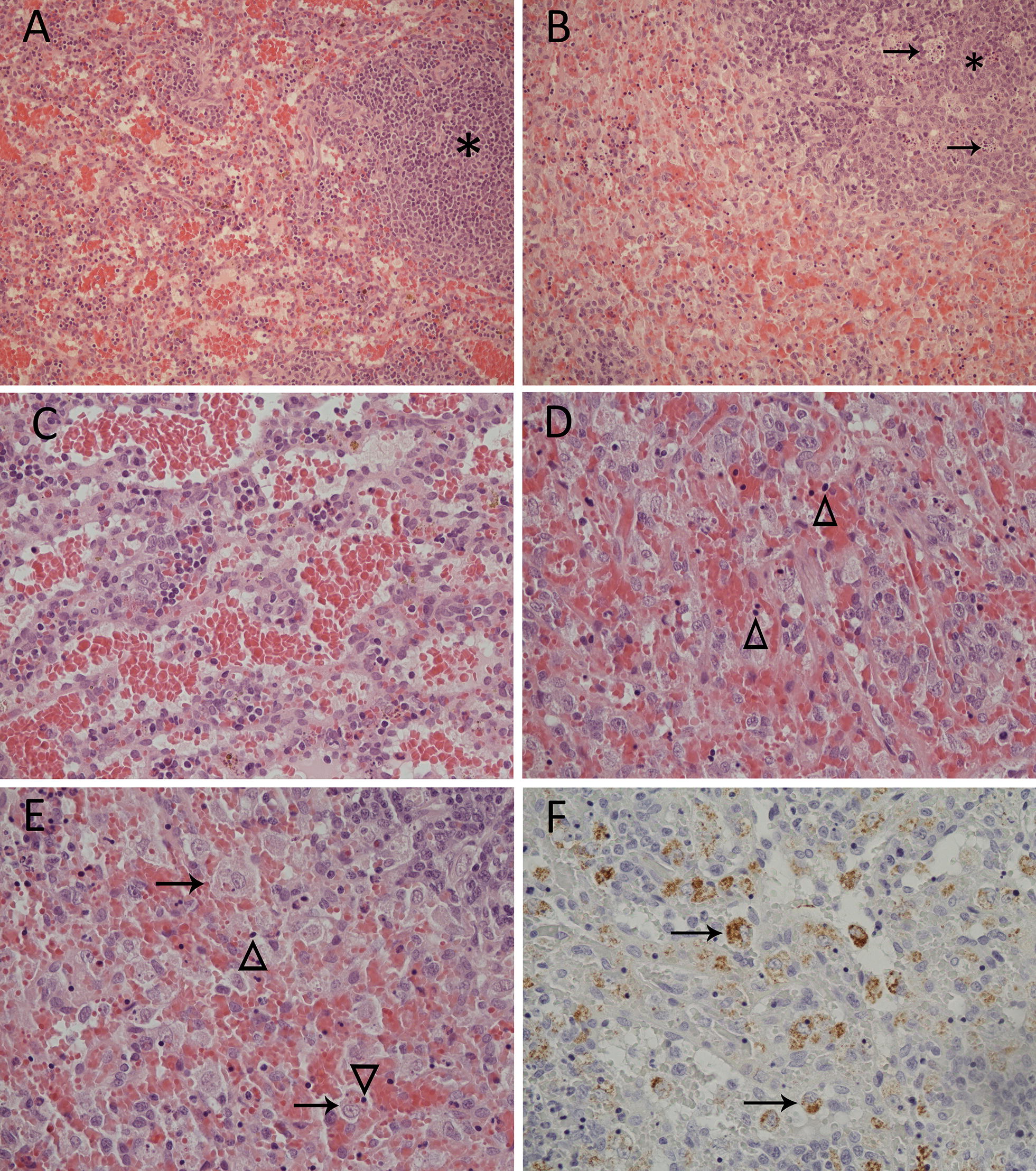
**Microscopic lesions and viral antigen localization in the spleen of a 5** **week old rabbit (*Oryctolagus cuniculus*) (rabbit 306) inoculated with** ***Lagovirus europaeus*** **G1.2 (BlMt-1).** Asterisks denote the white pulp. **A** Uninfected control (rabbit 130). 200× magnification. **B** Terminal stage of acute infection. There is lymphoid depletion and tingible body macrophages (arrows) in the white pulp and macrophage hyperplasia in the adjacent red pulp. 200× magnification. **C** Red pulp of the uninfected control (rabbit 130). 400× magnification. **D** Red pulp of rabbit 306. Note the indistinct, hyaline stroma and presence of small, dark pyknotic cells (arrowheads). 400× magnification. **E** Rabbit 306. Active phagocytosis by macrophages in the red pulp (arrows) and numerous pyknotic cells (arrowheads). The macrophage indicated at the bottom of the image has phagocytized a pyknotic cell. 400× magnification. **F** Immunohistochemical visualization of viral capsid antigen (brown staining) in the cytoplasm of viable macrophages (arrows) in the terminal stage of acute infection. Note that no staining is associated with pyknotic cells. 400× magnification.

The spleen of all animals was moderately to severely congested and abundant, activated macrophages (macrophage hyperplasia) displaying active phagocytosis were seen throughout the red pulp (Figure 2). In rabbits that died later (282, 303, 306), the stroma of the red pulp was hyaline, there was loss of cellular detail and numerous unidentifiable pyknotic cells were seen (Figure 2). Aggregates of amorphous eosinophilic material consistent with fibrin was seen scattered in the red pulp of rabbit 303. In the white pulp, there was moderate to severe decrease in the proportion of lymphocytes to macrophages reflecting general lymphoid depletion in comparison with the control animal. Active, mild to moderate lymphocytolysis was observed, and within the kittens, frequent tingible body macrophages were seen within the white pulp (Figure 2).

**Figure 3:**
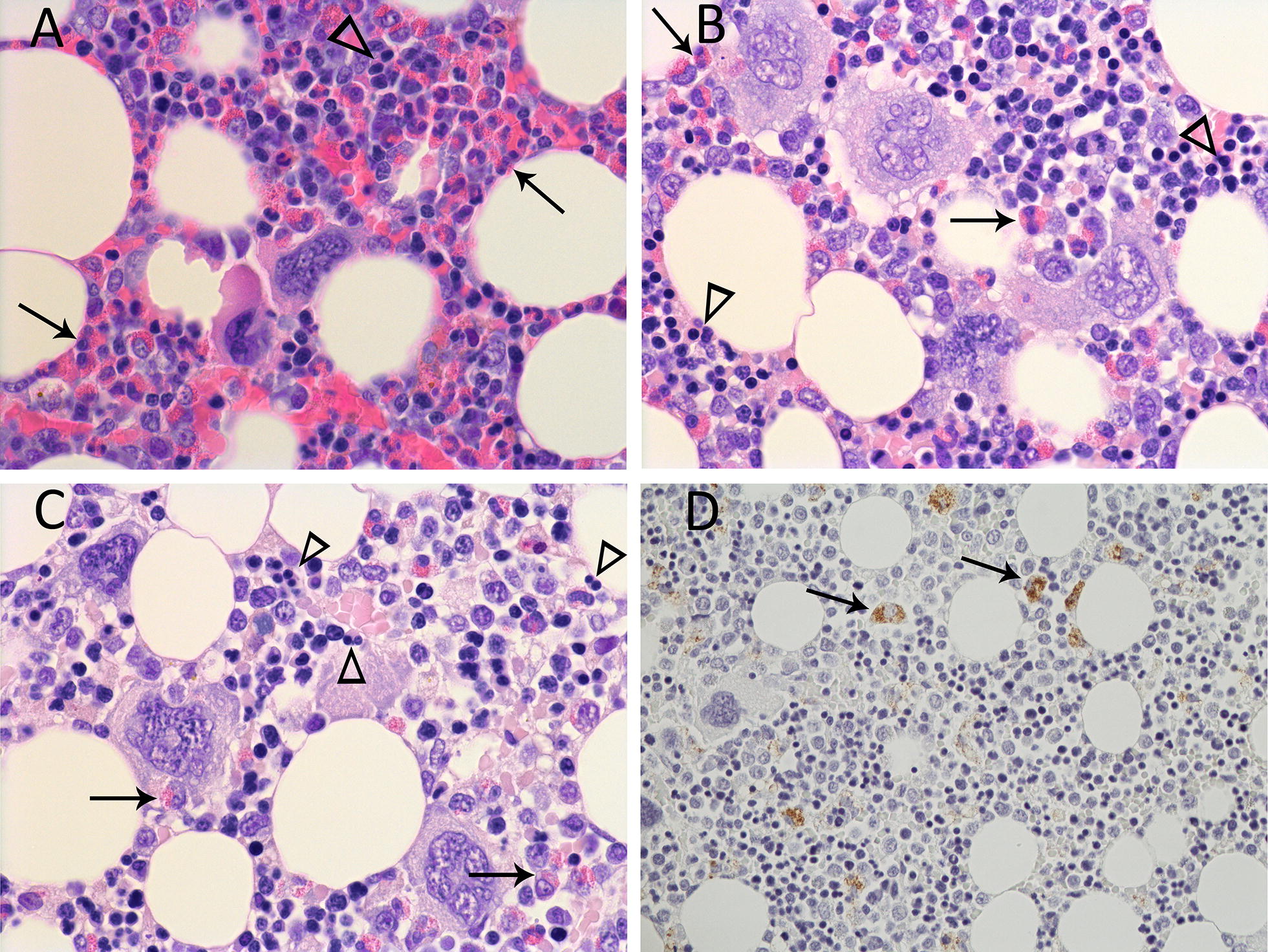
**Microscopic lesions and viral antigen localization in the bone marrow of adult rabbits (*Oryctolagus cuniculus*) inoculated with** ***Lagovirus europaeus*** **G1.2 (BlMt-1).** Arrows denote myeloid cells and arrowheads denote erythroid cells. **A** Uninfected control (rabbit 130). 600× magnification. **B** Bone marrow in an earlier stage of acute infection (rabbit 283). Note the decreasing proportion of myeloid to erythroid cells. 600× magnification. **C** Bone marrow at the terminal stage of acute infection (rabbit 282). Myeloid cells are scarce and erythroid cells at arrowheads are binucleated. 600× magnification. **D** Immunohistochemical visualization of viral capsid antigen (brown staining) in the cytoplasm of viable macrophages (arrows) in the terminal stage of acute infection (rabbit 282). 400× magnification.

Bone marrow was only available for examination from the adult rabbits and control animal. Similar to liver and spleen, changes in the bone marrow were more marked in the rabbit that was euthanized later following inoculation (282) (Figure 3). The M:E decreased and the proportion of immature myeloid cells increased (Table 3). In addition to the marked decrease in the proportion of myeloid to erythroid cells in rabbit 282, binucleated and even multinucleated rubricytes that typically are indicative of disrupted erythropoiesis were commonly seen (Figure 3).

**Table 3:** **Estimates of the myeloid-to-erythroid ratio and relative proportions of early stage myeloid cells versus later stage myeloid cells in the bone marrow of adult rabbits (** ***Oryctolagus cuniculus*** **) inoculated with** ***Lagovirus europaeus*** **GI.2 (BlMt-1)**

Rabbit	Myeloid-to-erythroid ratio	Proportion of early (myeloblasts, promyelocytes and myelocytes) versus later stage myeloid cells
130 (control)	2.08:1	16% versus 84%
283 (euthanized at 54 hpi)	0.99:1	24% versus 76%
282 (euthanized at 96 hpi)	0.47:1	55% versus 45%

Within other lymphoid tissues (Peyer’s patches, sacculus rotundus, cecal appendix, mesenteric lymph node, thymus), infected animals exhibited variable degrees of lymphocytolysis, presence of tingible body macrophages and lymphoid depletion. Again, whereas lesions were only mild to moderate in 283, they were similar or more severe in 282 and 303. No other lymphoid tissues were available for examination from animal 306. The thymus of rabbit 282 exhibited the most severe changes with marked, extensive lymphocytolysis and abundant tingible body macrophages containing apoptotic cellular and nuclear debris.

Pulmonary lesions were only seen in two of the four rabbits. In rabbit 282, moderate pulmonary congestion and oedema were accompanied by mildly increased numbers of plump intra-alveolar macrophages. In the lungs of the kitten that died spontaneously (303), infrequent fibrin thrombi were seen in small vessels and alveolar capillaries. Scattered to occasional pyknotic cells with hypereosinophilic cytoplasm similar to those observed in the spleen were seen in alveolar septae of 282, 303 and 306. Trachea was only available from the two adult animals and moderate submucosal congestion of the trachea was seen in both.

Renal lesions were confined to the kittens. Glomerular tufts were severely congested, occasionally accompanied by haemorrhage, and frequent fibrin thrombi were seen both in glomerular capillaries and adjacent small vessels. In scattered tubules, epithelium was either attenuated or plump and regenerative, indicative of acute tubular nephrosis.

With the exception of rare thrombi seen in small vessels of the myocardium of rabbit 303, no other significant lesions were seen in the other tissues examined for all animals.

### Immunohistochemistry

**Figure 4:**
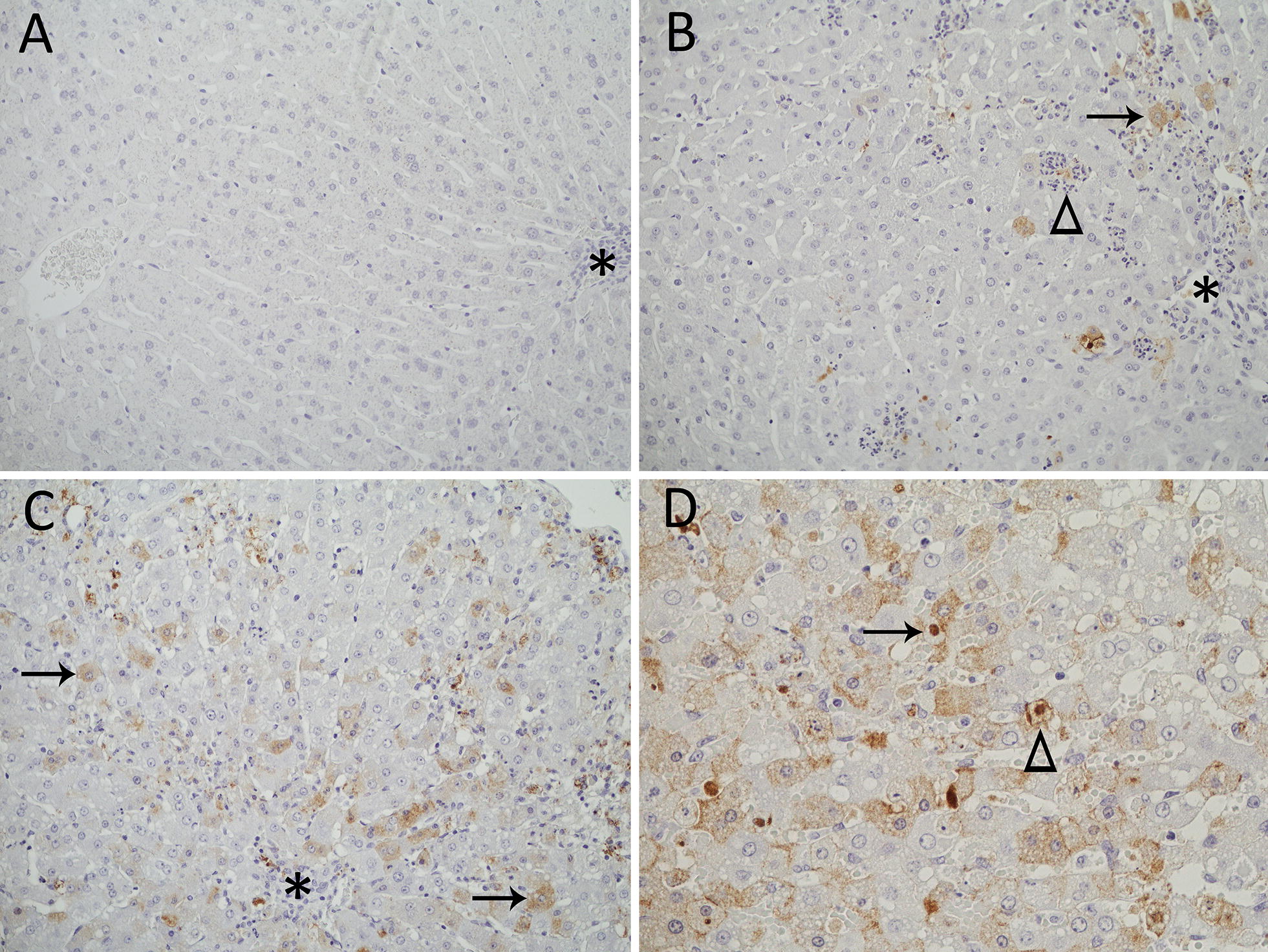
**Viral antigen localization in the liver of rabbits (*Oryctolagus cuniculus*) inoculated with** ***Lagovirus europaeus*** **G1.2 (BlMt-1).** Asterisks denote portal areas. **A** Uninfected control (rabbit 130). No antigen (brown staining) is seen. 200× magnification. **B** Earlier stage of acute infection in an adult rabbit (rabbit 283). A small number of periportal hepatocytes contain viral antigen (brown staining, arrow). A cluster of heterophils surrounds necrotic, antigen-containing debris interpreted to be the remnants of an infected hepatocyte (arrowhead). 200× magnification. **C** Terminal stage of acute infection in an adult rabbit (rabbit 282). There is cytoplasmic staining in hepatocytes throughout the lobule (arrows). 200× magnification. **D** Terminal stage of acute infection in a 5 week old rabbit (rabbit 303). In addition to the finely stippled intracytoplasmic staining of infected hepatocytes, there is occasional intranuclear staining (arrow, arrowhead) and staining of the cell membrane (arrowhead). 400× magnification.

Viral antigen was visualized in the liver of all four rabbits and for animal 283, the liver was the only organ in which viral antigen was detected. In general, viral antigen was predominantly found within normal and degenerate hepatocytes, where staining typically was finely stippled and could be seen throughout the cytoplasm and/or concentrated around the periphery at the cell membrane (Figure 4). Infrequently, intracytoplasmic staining was confined to a focal area adjacent to the nucleus. Intranuclear staining, although it occurred, was rare (Figure 4). Rare to occasional Kupffer cells also contained viral antigen. Within Kupffer cells, staining was always intracytoplasmic and coarsely clumped. Additionally, coarsely clumped antigen often could be seen associated with hepatocellular remnants and inflammatory cell infiltrates, but exact localization could not be readily discerned. In animals 282, 303 and 306, rare to infrequent intravascular leukocytes morphologically consistent with monocytes or macrophages showed coarsely clumped intracytoplasmic staining. Biliary epithelium and vascular endothelium did not contain detectable viral antigen.

Within the liver, the amount of viral antigen observed and its distribution varied between rabbits. Rabbit 283 contained the least amount of viral antigen and staining was confined primarily to hepatocytes and hepatocellular remnants admixed with heterophil infiltration within periportal areas (Figure 4). The other three rabbits contained markedly more viral antigen, but whereas it was primarily located within hepatocytes in the form of finely stippled stain throughout the lobule in rabbits 282 and 303, in rabbit 306, antigen was more typically coarsely clumped and distributed throughout the lobule within hepatocytes, Kupffer cells and within inflammatory and necrotic debris.

Within the spleen of rabbits 282, 303 and 306, coarsely clumped viral antigen was seen within the cytoplasm of frequent (282) to abundant (303, 306) macrophages (Figure 2). Antigen-containing macrophages were numerous and conspicuous at the marginal zone of the white pulp and frequently within sinus histiocytes of the red pulp (Figure 2). They also occasionally contained intracytoplasmic, basophilic, round, pyknotic bodies similar in appearance to the unidentified pyknotic cells in the red pulp. Otherwise, the majority of pyknotic cells were not associated with viral antigen (Figure 2). No antigen was associated with lymphocytolysis or observed within tingible body macrophages of the white pulp. No antigen was observed in the spleen of rabbit 283.

Similar to the spleen, occasional to more frequent macrophages in the bone marrow of rabbit 282 contained coarsely clumped, intracytoplasmic viral antigen (Figure 3). Antigen-containing macrophages showed no evidence of degeneration or cell death. Megakaryocytes and granulocytic and erythroid cell lines were devoid of antigen. No viral antigen was seen in the bone marrow of rabbit 283 and bone marrow was unavailable for examination in the kittens.

A small amount of viral antigen was detected within the lungs of rabbits 282, 303 and 306. Staining primarily was confined to the alveolar septae, both clearly cell-associated as clumped intracytoplasmic material within cells morphologically consistent with macrophages, or within septal capillaries, often associated with material consistent with fibrin. Scattered leukocytes with large, open nuclei and abundant cytoplasm (probable monocytes or macrophages) within larger vessels also contained clumped intracytoplasmic antigen. No antigen was detected within alveolar macrophages nor was antigen observed associated with pyknotic cells seen within alveolar septae.

No viral antigen was detected in the kidneys of the two adults 283 and 282. In contrast, both kittens had a small amount of detectable antigen which was seen associated with fibrin thrombi within glomeruli and small adjacent vessels. Rare leukocytes within larger vessels contained intracytoplasmic antigen. Antigen was not detected in tubular epithelium.

Viral antigen was not detected in any other tissues, including those that displayed microscopic lesions. No antigen was seen associated with lymphocytes, lymphocytolysis, tinigible body macrophages and/or lymphoid depletion in lymphoid tissues. Likewise, no staining was seen within the heart of 303 or the congested trachea of 282 and 283. Viral antigen was never detected in any tissues of the control animal 130.

### Molecular analyses

Viral RNA was detected by RT-qPCR in all tissues analysed and viral loads (capsid gene copies per mg tissue) are presented in Table 4. For the majority of tissues analysed, Rabbit 283, the animal euthanized earliest after inoculation, had the lowest viral load, often by an order of magnitude or more. Serum levels of virus in this rabbit were 3–4 orders of magnitude lower than in other animals. Highest viral loads were detected in the serum (except for animal 283) and liver, followed by the spleen.

**Table 4:** **Viral copies of** ***Lagovirus europaeus*** **GI.2 (BlMt-1) per milligram tissue calculated from RT-qPCR analyses**

Rabbit	Adults		5 week old kittens				
	282	283	302	303	304	305	306
Liver (hilar)	2.42E+08	1.64E+08	n.c.	n.c.	n.c.	n.c.	n.c.
Liver (distal)	3.03E+08	1.33E+08	n.c.	n.c.	n.c.	n.c.	n.c.
Liver (not specified)	n.c.	n.c.	4.67E+08	6.16E+08	3.56E+08	9.01E+08	5.07E+08
Bile	1.46E+06	3.26E+06	n.c.	n.c.	n.c.	n.c.	n.c.
Spleen	1.17E+08	2.36E+07	1.91E+08	1.02E+08	4.08E+07	8.55E+07	3.23E+08
Lung	7.42E+07	2.38E+06	1.30E+08	4.39E+07	9.94E+07	2.00E+08	2.77E+07
Heart	3.27E+06	1.02E+06	2.22E+07	2.16E+07	7.52E+06	n.c.	1.43E+07
Kidney	3.22E+06	6.76E+05	1.30E+07	3.91E+07	6.16E+06	1.20E+07	1.75E+07
Thymus	1.29E+06	1.15E+05	n.c.	n.c.	n.c.	n.c.	n.c.
Mesenteric lymph node	n.c.	4.24E+06	3.00E+06	n.c.	1.84E+06	n.c.	6.04E+06
Duodenum	5.80E+05	1.27E+06	3.23E+06	2.34E+06	2.61E+06	2.23E+06	1.47E+06
Jejunum	1.75E+06	3.64E+06	4.07E+06	3.70E+06	1.83E+06	5.15E+06	2.73E+06
Ileum	2.84E+06	3.96E+03	1.50E+06	1.26E+06	1.78E+06	1.87E+06	2.20E+06
Peyers patches	8.38E+05	1.25E+06	3.51E+06	1.40E+06	1.66E+06	7.12E+05	3.53E+06
Appendix	5.35E+05	1.12E+04	9.95E+05	6.78E+05	7.63E+05	8.16E+05	7.23E+05
Sacculus rotundus	4.74E+05	7.18E+04	n.c.	n.c.	n.c.	n.c.	n.c.
Cecum	3.63E+05	4.98E+04	n.c.	n.c.	n.c.	n.c.	n.c.
Colon (+contents)	1.50E+06	n.c.	1.34E+06	2.95E+05	5.24E+05	1.21E+06	3.12E+05
Trachea	2.27E+07	n.c.	n.c.	n.c.	n.c.	n.c.	n.c.
Bone marrow from femur	1.58E+07	3.85E+05	n.c.	n.c.	n.c.	n.c.	n.c.
Brain	3.95E+06	2.55E+04	n.c.	n.c.	n.c.	n.c.	n.c.
Buffy coat	5.35E+06	2.13E+04	n.c.	n.c.	n.c.	n.c.	n.c.
Serum	1.29E+08	2.21E+05	2.42E+09	3.87E+08	2.43E+08	n.c.	7.42E+08

The analysis was done in technical duplicate per tissue and values represent the mean.

n.c.: sample not collected.

## Discussion

In this study, clinical signs, disease and case fatality rate caused by the Australian strain of *L. europaeus* GI.2 closely mimicked that of classic RHD in adult rabbits caused by *L. europaeus* GI.1 strains [1]. Death or severe disease necessitating euthanasia occurred within 96 hpi for all animals, with an average time to death or euthanasia of 73 hpi. All animals developed a fever within 52 hpi and, with the exception of 282, either died suddenly or were depressed, hypothermic and/or anorexic prior to euthanasia or death. This is compatible with the peracute and acute forms of RHD observed in GI.1 infected rabbits [31, 32]. Although rabbit 282 was behaving normally up to 96 hpi, there were widespread liver lesions, viral loads in the serum and liver were very high and body weight had dropped more than 10%. It is not possible to predict whether this animal ultimately would have survived or succumbed to subacute RHD, but this variation in disease progression and mortality rate among animals is consistent with that seen in classic RHD caused by GI.1 and differs from the longer disease course and lower case fatality rates described for early strains of GI.2 [2]. Although the number of animals used in this study was small, our findings strongly suggest that BlMt-1 is much more pathogenic than earlier reported strains of GI.2 that were detected in Europe [2]. This is in line with findings by Capucci et al. [21] who carried out experimental infections of a small number of domestic rabbits with more recent European GI.2 isolates, and similarly report very high case fatality rates. The European virus most closely related to BlMt-1, a recombinant GI.1bP–GI.2 from 2014, has reportedly replaced previously circulating GI.1 strains [12] and has led to substantial declines in wild rabbit numbers in the Iberian Peninsula [33], indicative of a highly virulent virus. Capucci et al. [21] hypothesized that GI.2 strains have evolved in their natural hosts since their emergence in 2010, and similar to what was proposed for GI.1 strains, selection pressure favours strains with higher pathogenicity [34]. Our data from this more recent isolate (BlMt-1) is consistent with this hypothesis.

Consistent with all previous studies on RHD, the liver is the target organ of BlMt-1. Based on time of euthanasia after inoculation, lower viral tissues loads (Table 4) and data from previous studies on GI.1 viruses (e.g. [35, 36]), rabbit 283 represents an earlier stage of disease than rabbits 282, 303 and 306. By comparing results from these animals, disease progression mimics that of other pathogenic lagoviruses. Infection in the liver began with degeneration and death of periportal hepatocytes at and adjacent to the limiting plate and incited a marked heterophilic inflammatory response. As disease progressed, affected hepatocytes became more numerous and widespread throughout the lobule, although centrilobular hepatocytes still tended to be spared, reflecting disease advancement from the periphery of lobules centrally. Occasional to moderate numbers of heterophils were still present in the liver and often associated with dead hepatocytes, but they were distributed throughout the lobule rather than confined to periportal regions. These lesions were coupled with activation and hyperplasia of Kupffer cells. The presence of bile duct hyperplasia, mild mononuclear inflammation and mild fibrosis portally in all rabbits, including the control, was interpreted to be unrelated to RHD. Rather, this has been described as an incidental finding in normal rabbits [37] or may reflect previous, unrelated insult. To conclude, BlMt-1 induces the same lesions in both adults and kittens as reported for GI.1 viruses in adults.

In contrast to GI.1 strains, both Le Gall-Reculé et al. [2] and Dalton et al. [13] report mortality rates of up to 50% for kittens that were involved in RHD mortality events caused by GI.2. Although sample size in this study is small, all five inoculated kittens succumbed to disease. Four died and the fifth was euthanized. The fifth kitten had severe, extensive liver lesions deemed incompatible with survival, supporting a case fatality rate of 5/5 kittens for BlMt-1 in this study. Disease course was acute, with all kittens dying or euthanized by 90 hpi. This differs from GI.1 viruses where infected kittens of this age do not develop clinical disease, although the reasons are not fully understood. Viral receptor expression, innate immunity and changes in liver function around 5–6 weeks of age have all been suggested to play a role [38–40]. One very clear finding of this study was that inflammation in the liver in both BlMt-1 inoculated adults and kittens was similar and heterophilic. This is not the case for GI.1, where adults display heterophilic inflammation throughout the disease course, but kittens develop lymphocytic inflammation by 48 hpi [41]. Mikami et al. [42] also showed that inflammation, predominantly lymphocytes admixed with macrophages and some heterophils continued to increase up to 96 hpi. Ferreira et al. [41] further hypothesize that these lymphocytes seen in infected kittens respond to surface presentation of lagovirus antigen on hepatocytes and that this is important in modulating resistance to development of clinical disease. Lymphocytic inflammation was not a feature in kittens succumbing to BlMt-1. This suggests that GI.2 viruses may have the ability to replicate within hepatocytes without invoking this postulated protective lymphocytic inflammatory response. Likewise, the other two proposed mechanisms of resistance to GI.1 strains in kittens apparently were overcome and investigation into underlying mechanisms would provide critical insight into pathogenicity determinants in lagoviruses.

Apoptotic programmed cell death is a common feature in viral hepatitides (e.g. [43, 44]) and has been shown to occur in RHD caused by GI.1 strains [45–47]. Hepatic lesions in this study showed morphologic features of both apoptosis and necrosis. Earlier in infection (rabbit 283), dead hepatocytes commonly display features of apoptosis: shrinkage, hypereosinophilia, karyorrhexis and pyknotic nuclear remnants, and rounding up and dissociation of cells from the basement membrane [30]. However, the intense, accompanying heterophilic inflammation is not a feature typically associated with apoptosis. Likewise, there also are foci of lytic necrosis in areas of the inflammation, and in later stages of infection, single cells with morphology more consistent with single cell necrosis (e.g. increased cell size, vacuolation, loss of cellular detail, karyolysis) are seen. Overlap of apoptosis and necrosis within the same disease process is well-recognized and may occur, for example, when phagocytic capacity to clear apoptotic remnants is overwhelmed [44]. Additionally, our current understanding of programmed cell death is rapidly expanding and a host of newly recognized mechanisms of programmed cell death involving inflammation (e.g. necroptosis, NETosis, pyroptosis) have been described and can play critical roles in antiviral immunity, particularly when viral mechanisms block apoptosis [48–50]. RHD has already been proposed as a model for acute fulminant liver disease in humans [51] and continued investigations into the cell death mechanisms in RHD may provide insight into the pathogenesis of a wider range of liver diseases in different species.

In extrahepatic tissues, lesions included thrombi and haemorrhage typical of disseminated intravascular coagulation (DIC) [52], splenic necrosis, lymphocytolysis and lymphoid depletion. Within the bone marrow, there was decreased M:E, increased proportion of immature myeloid cells and presence of atypical rubricytes. Both DIC and lymphoid depletion have been described in GI.1 infections [1, 31, 36], but bone marrow changes have not yet been described for RHD and provided additional insight into pathogenesis. In the adult rabbit that survived longer (282), the proportion of heterophils and myeloid precursors decreased and of the myeloid cells remaining, the proportion of mature stages decreased. This left shift in the bone marrow is consistent with the heteropenia described in RHD by Ferreira et al. [53]. These authors proposed three mechanisms to account for the heteropenia: sequestration of mature heterophils, cytotoxic effects of the virus on circulating heterophils and cytotoxic effects on granulocytic myeloid precursors in the bone marrow. The moderate to marked heterophilic inflammation seen in the liver, particularly earlier in disease, favours the first hypothesis regarding sequestration and consumption of mature heterophils in the liver. While cytotoxic effects of the virus on circulating heterophils or myeloid precursors cannot be excluded, the absence of viral antigen within circulating heterophils or granulocytic precursors in the bone marrow does not support direct viral cytotoxicity. M:E is used to detect variation in rates of differentiation of myeloid and erythroid precursors. Although M:E for the control animal 130 was higher than the reported mean of approximately 1.1:1 for laboratory rabbits [54], the comparisons presented here are relative and are used as a tool to compare animals within this rabbit colony. The frequent bi- and multinucleate rubricytes may represent erythroid dysplasia or dyserythropoiesis. While acquired dyserythropoiesis most often is associated with nutritional deficiencies [55], chemotherapeutic drugs and myeloid neoplasms [56], dysplastic changes have also been reported in certain viral diseases such as those caused by Feline immunodeficiency virus [57] and Feline Leukemia Virus [58]. Bone marrow evaluation using formalin-fixed material has limitations regarding more detailed discrimination of cells. Therefore, further investigation, including concurrent evaluation of haematology and bone marrow cytology in rabbits with RHD, is required to better define the myeloid hypoplasia and left shift, and to confirm and understand the underlying mechanisms of the apparent erythroid dysplasia in RHD. In contrast to findings by Dalton et al. [5], no lesions were seen in the villi of the small intestine of kittens, nor were they seen in the adults.

Using immunohistochemistry, presence of viral antigen was clearly associated with hepatic lesions and degenerate and dead hepatocytes and antigen spread from periportal areas towards the central vein as disease progressed. Location of virus within hepatocytes was primarily intracytoplasmic and finely stippled, although rimming of the cell membrane, as described by Park and Itakura [59] for GI.1, was also seen (Figure 4). These authors suggest that the different staining patterns reflect different stages in infection as is observed in other viruses. Lagoviruses are thought to replicate within the cytoplasm [1], but consistent with other reports [42, 59, 60], infrequent antigen was also seen within the nucleus (Figure 4). The presence of viral capsid protein within the nucleus remains unexplained. However, as hypothesized by Ramiro-Ibanez et al. [61], because so little is known about lagovirus replication, localization of the viral capsid within the nucleus may be required for some stage in the virus life cycle.

In addition to hepatocytes, viral antigen was also detected within cytoplasm of cells of the mononuclear phagocytic system. These include Kupffer cells in the liver, macrophages within the spleen, bone marrow and lungs, as well as cells consistent with intravascular monocytes or even tissue macrophages that have re-entered circulation in various tissues. While functions of these cells are diverse, they are phagocytic and can play a role in both innate and adaptive immunity [62]. Within these cells, staining was dark and coarsely clumped similar to hepatocyte remnants in areas of lytic necrosis in the liver, rather than finely stippled as seen within hepatocytes. Additionally, whereas hepatocytes showed cytopathic effects from the virus, the phagocytic cells showed no signs of degeneration or death in this study. In these macrophages, antigen staining patterns may therefore be more compatible with phagocytized material rather than replicating virus. However, Kimura et al. [63] show evidence for viral replication within macrophages using in situ hybridization and further investigation into the role of macrophages in RHD, including spread from the site of entry to the liver, is needed.

Within lymphoid tissues, viral antigen was not present in lymphocytes or associated with lymphocytolysis or lymphoid depletion. This suggests that lymphocytolysis is the result of a secondary mechanism, for example increased glucocorticoids associated with stress as is seen in lymphoid tissues, especially the thymus [64].

Assessment of immunohistochemical staining of inoculated animals in conjunction with both the negative tissue and immunoglobulin controls allowed us to conclude that the immunohistochemistry performed on this material was very specific and that non-specific staining and background staining were absent. However, the analysis required a large amount of virus to be present before antigen could be detected in situ. Antigen was readily detected in tissues with 10^8^ viral copies per mg and in just over half of the tissues containing 10^7^ viral copies per mg tissue. However, 10^6^ viral copies per mg tissue was below the detection limit for the immunohistochemical analysis of this material. We are confident that staining, when present, corresponds to viral antigen, but we cannot exclude the possibility that tissues or cells containing lower amounts of virus were not detected with immunohistochemistry. While PCR is more sensitive, it does not allow for specific in situ localization of virus within tissues nor can it be correlated with microscopic lesions. Using immunohistochemistry, intracellular virus presence was seen in liver and in cells of the mononuclear phagocytic system in the spleen, bone marrow, lungs and intravascular monocytes. However, virus was also detected in high levels in the serum and in reasonable amounts (10^6^ copies per mg) in numerous other tissues, including trachea and small intestine. The lack of lesions in these tissues suggests PCR is likely detecting viral RNA in free viral particles in the blood in these tissues, as well as in the lumen of the intestine from virus excreted via the bile, respectively. Additionally, the high viral load in bone marrow may contribute to the epidemiology of RHD in wild rabbit populations by prolonging environmental persistence of virus when all other tissues except for the skeleton are gone, provided the virus material in bone marrow remains infectious.

To conclude, unlike early GI.2 viruses [2], the pathogenicity of the 2015 BlMt-1 strain used in this study is comparable to that of GI.1 strains in adult rabbits. Additionally, and in contrast to GI.1, it is as pathogenic for kittens as adults. The inflammatory response in the liver of kittens inoculated with BlMt-1 differs from that reported from kittens infected with GI.1 strains, providing support for the proposed role of lymphocytes in modulating resistance to clinical disease from GI.1 infection. The strong susceptibility of kittens to disease by the BlMt-1 virus adds a new dimension to the epidemiology of RHD in wild rabbit populations in Australia and will likely have implications for rabbit biocontrol in this country. A recent study reports that the GI.1bP–GI.2 virus (to which BlMt-1 belongs) has replaced endemic GI.1 strains within 18 months of its arrival in Australia [10]. Mahar et al. [10] further hypothesize that the ability of this virus to infect and kill rabbits at a much younger age provides a key competitive advantage in the field, as new cohorts of rabbits become susceptible to lethal infection at a much earlier age compared to GI.1. Indeed, for 17 of the viruses sequenced in their study, the body mass of the deceased rabbits was known, and eight of these isolates (47%) were from kittens weighing less than 600 g, corresponding to 4–8 weeks of age. The differences in pathogenicity between GI.2 strains, coupled with differences in susceptible age groups and host species when compared to GI.1 viruses, provide an excellent opportunity to explore the genetic and immunological determinants of pathogenicity in lagoviruses.

## Abbreviations

RHDV: rabbit haemorrhagic disease virus
RHD: rabbit haemorrhagic disease
hpi: hours post-inoculation
M:E: myeloid-to-erythroid ratio
RT-qPCR: reverse transcriptase quantitative polymerase chain reaction
